# Case Report: Hybrid closed-loop insulin pump can significantly improve awareness of hypoglycemia

**DOI:** 10.3389/fcdhc.2025.1536038

**Published:** 2025-02-21

**Authors:** Eliška Zadáková, Eva Horová

**Affiliations:** 3_rd_ Department of Internal Medicine, General University Hospital and 1_st_ Faculty of Medicine, Charles University, Prague, Czechia

**Keywords:** diabetes type 1, hypoglycemia, hypoglycemia unawareness syndrome, hybrid closed-loop insulin pump, case report

## Abstract

Impaired awareness of hypoglycemia remains an issue even in the era of modern technologies, as patients with type 1 diabetes (T1DM) face stricter requirements for glycemic targets. The evaluation of hypoglycemia awareness can be accomplished using questionnaires (Clarke and Gold scores) in combination with clinical appearance and sensor data. A 45-year-old man with T1DM was referred to our clinic in July 2019 due to impaired hypoglycemia awareness and repeated severe hypoglycemic episodes resulting in unconsciousness. At that time, he was driving both a car and a motorcycle. Despite good compliance, increased target values and implementation of continuous glucose monitoring (CGM) with alarms, prolonged hypoglycemias were not eliminated. Therefore, the patient was referred for pancreatic islet transplantation, but he decided not to undergo. In May 2021, his driving license was suspended, which eventually led him to accept treatment with a hybrid closed-loop insulin pump (AID). Shortly after initiation, he achieved satisfactory glycemic control, reduced time spent in hypoglycemia, and had no severe hypoglycemic episodes. According to the questionnaires, the hypoglycemia awareness has improved and his driving license was reinstated. This case study highlights the critical importance of identifying impaired awareness of hypoglycemia, its potential social impacts, and the opportunities for using new technologies to reverse this complication.

## Introduction

Frequent episodes of hypoglycemia can lead to the development of impaired awareness of hypoglycemia, a condition in which a person with diabetes experiences an attenuation of early warning symptoms of low blood sugar. This can progress to hypoglycemia unawareness, where the patient does not experience the usual symptoms of hypoglycemia and is unable to recognize the occurrence of a hypoglycemic event (1). The prevalence of impaired awareness of hypoglycemia is approximately 25-30% in patients with T1D and is associated with a six-fold greater frequency of severe hypoglycemia (2). In addition to health problems, impaired awareness of hypoglycemia is also a socio-legal issue, since it can lead to driving license suspension.

This case is of interest because it offers a novel solution to the well-known problem of hypoglycemia unawareness, presenting it from a different perspective. It highlights the importance of evaluating and managing impaired awareness of hypoglycemia not only with the usual methods but also with new technologies. The approach of using a combination of Clarke and Gold score questionnaires alongside clinical appearance and sensor data analysis to assess impaired awareness of hypoglycemia is new in diabetology and relies on the widespread adoption of CGM among patients with diabetes. Before the advent of AID systems, pancreatic islet transplantation was the only solution for severe hypoglycemia unawareness. However, this case, along with studies on AID systems (3, 4), demonstrates that this serious complication can be both reversible and manageable using advanced technology, eliminating the need for transplantation.

With the continuous advancement and widespread adoption of new technologies, hypoglycemia unawareness may become increasingly rare in the future. This case also highlights the importance of recognizing that, in instances of hypoglycemia unawareness, it may sometimes be necessary to revoke a driving license in accordance with local laws and recommendations. This step is always challenging, but it can motivate patients to adopt the recommended treatment, which is beneficial for them and can lead to the resolution of the complication.

## Case description

A 45-year-old man (weight 82 kg, BMI 25.6 kg/m2) with T1DM since 1996 began attending our diabetology center in 2019. At that time, he was already diagnosed with impaired awareness of hypoglycemia and had a history of several severe hypoglycemic episodes with unconsciousness requiring emergency services. He was treated with insulin pens in multiple dose intensified regimen, using a combination of glargine 300 (28 IU) once daily and glulisine with main meals and for corrections (approximately 30 IU/day). Besides insulin, he did not take any medication. As part of standard care, he was regularly examined for diabetes complications. Due to non-proliferative diabetic retinopathy, ophthalmological examinations were conducted once a year. During each diabetology check-up, conducted every 3 to 4 months, laboratory parameters of kidney function were assessed from blood and urine samples, with results within normal ranges, and diabetic neuropathy was routinely screened. In 2020, he underwent a neurological examination with electromyography to exclude neuropathy, which showed negative results. From a social history perspective, he worked as a technical engineer, lived with his family and two young children, and regularly drove a car and motorcycle.

## Diagnostic assessment

**Figure 1:**
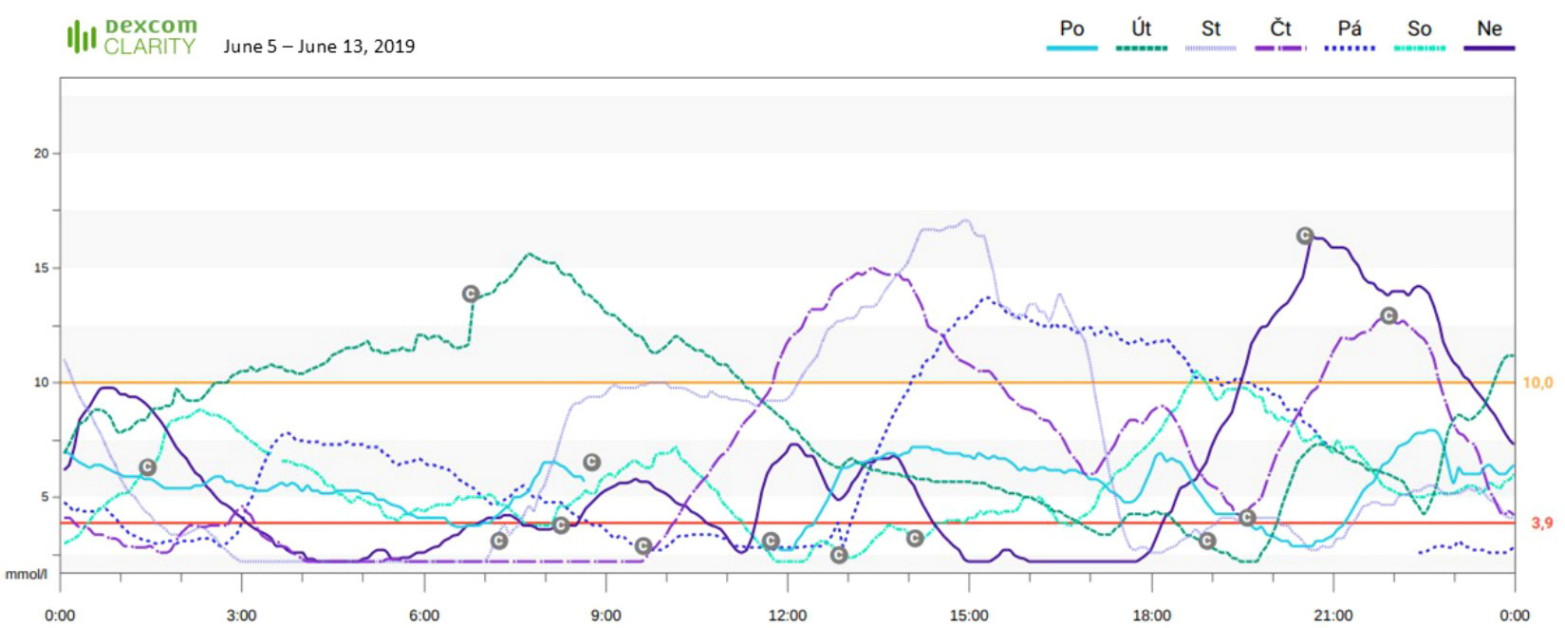
CGM glucose levels during one week in June 2019 showing severe prolonged nocturnal hypoglycemia, indicative of impaired hypoglycemia awareness. HbA1c was 7.1%, time in range (3.9-10.0 mmol/l, 70-180 mg/dL) 59%, time below range 23%.

**Figure 2:**
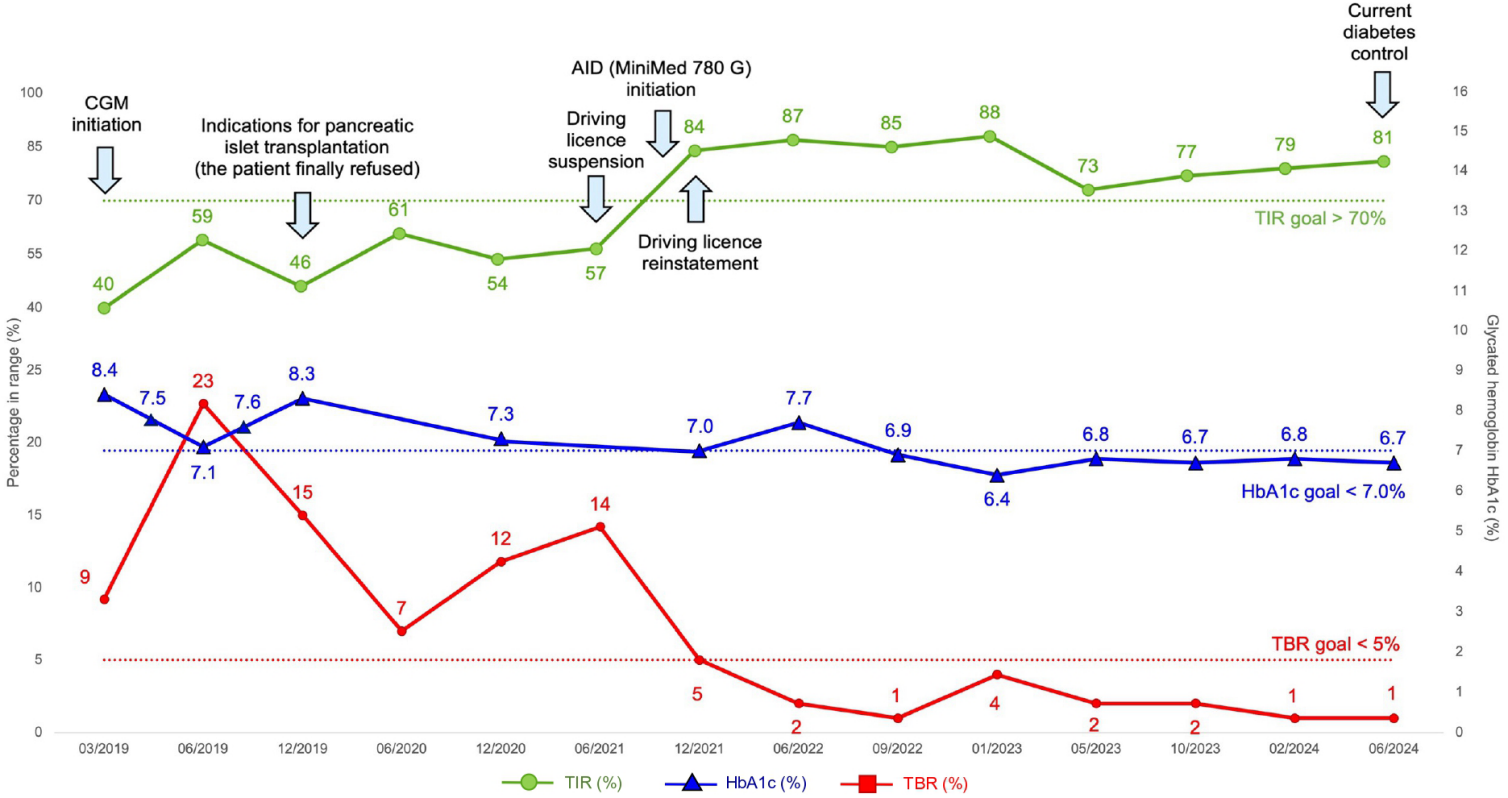
Timeline of diabetes control (HbA1c, TIR, TBR) development from January 2019 to present. AID, automated insulin delivery i.e. hybrid closed-loop insulin pump; CGM, continuous glucose monitoring; TBR, time below range; TIR, time in range.

The initial glycated hemoglobin (HbA1c) value in 2019 was 8.4% (68 mmol/mol). He was immediately provided with sensors with alarms (Dexcom G5 and G6) (5), which led to a significant decrease in HbA1c to 7.1% (54 mmol/mol) within the first few months. However, despite the patient’s efforts, there was an increase in the percentage of time spent in hypoglycemia (time below range, TBR) (**Figure 1**) and one new episode of severe hypoglycemia. The development of sensor parameters and HbA1c is shown in **Figure 2**. The patient completed validated questionnaires for assessing hypoglycemia awareness (Clarke score 6, Gold score 6) with the expected result of impaired awareness of hypoglycemia.

## Therapeutic intervention

The patient was compliant, had undergone repeated education with a nutrition therapist, was able to calculate carbohydrates, managed flexible insulin dosing, and used a bolus calculator (mySugr). We reduced his total daily dose (TDD) of insulin to approximately 52 IU/day, his carbohydrate-insulin ratio (CIR) was 8-10-8 g and insulin-sensitivity factor (ISF) (04-10 AM) 1.8 mmol/L (32.4 mg/dL) and (10-04 AM) 2.0 mmol/L (36 mg/dL). Despite higher target glucose values and use of CGM (alert settings: Low alert 4.4 or 5.0 mmol/L [80 or 90 mg/dL], Fall Rate on, Urgent Low Soon on, Snooze Time 15 minutes), prolonged hypoglycemia could not be eliminated during 2020, especially after physical activity. The patient admitted that he occasionally had to turn off or mute the alerts due to work or alarm fatigue. He was offered an AID system, but he declined and agreed to undergo pancreatic islet transplantation. However, after completing all preparatory examinations and receiving approval for the transplant, the patient ultimately declined due to the ongoing COVID-19 pandemic and concerns about immunosuppression.

Based on the clinical manifestations, high percentage of TBR, Clarke and Gold score questionnaire results, and the consensus of diabetologists, the patient’s driving licence was suspended in April 2021 according to local legislation. After several months, this led the patient to agree to initiate treatment with the AID system MiniMed 780 G (insulin Ultra-Rapid Lispro, initial settings; Basal 1: 0:00-24:00 0.85 IU/h i.e. 20.4 IU/day, Basal 2: 00:00-05:00 0.65 IU/h, 05:00-11:00 0.7 IU/h, 11:00-22:00 0.85 IU/h, 22:00-24:00 0.7 IU/h i.e. 18.2 IU/day, Bolus Wizard on, CIR 8-10-8 g, ISF 1.8 mmol/l [32.4 mg/dL], blood glucose target 6.0-6.5 mmol/L [108–117 mg/dL], active insulin time 2:30, SmartGuard target glucose 6.7 mmol/L [120 mg/dL], alerts; Before Low 4.6 mmol/L [82.8 mg/dL], Low 4.2 mmol/L [75.6 mg/dL], Before High 9.5 mmol/L [171 mg/dL], High 11.0 mmol/L [198 mg/dL]. After SmartGuard initiation, the TDD averaged 54 IU/day, and the Carbs entered averaged 200 g/day).

## Follow-up and outcomes

**Figure 3:**
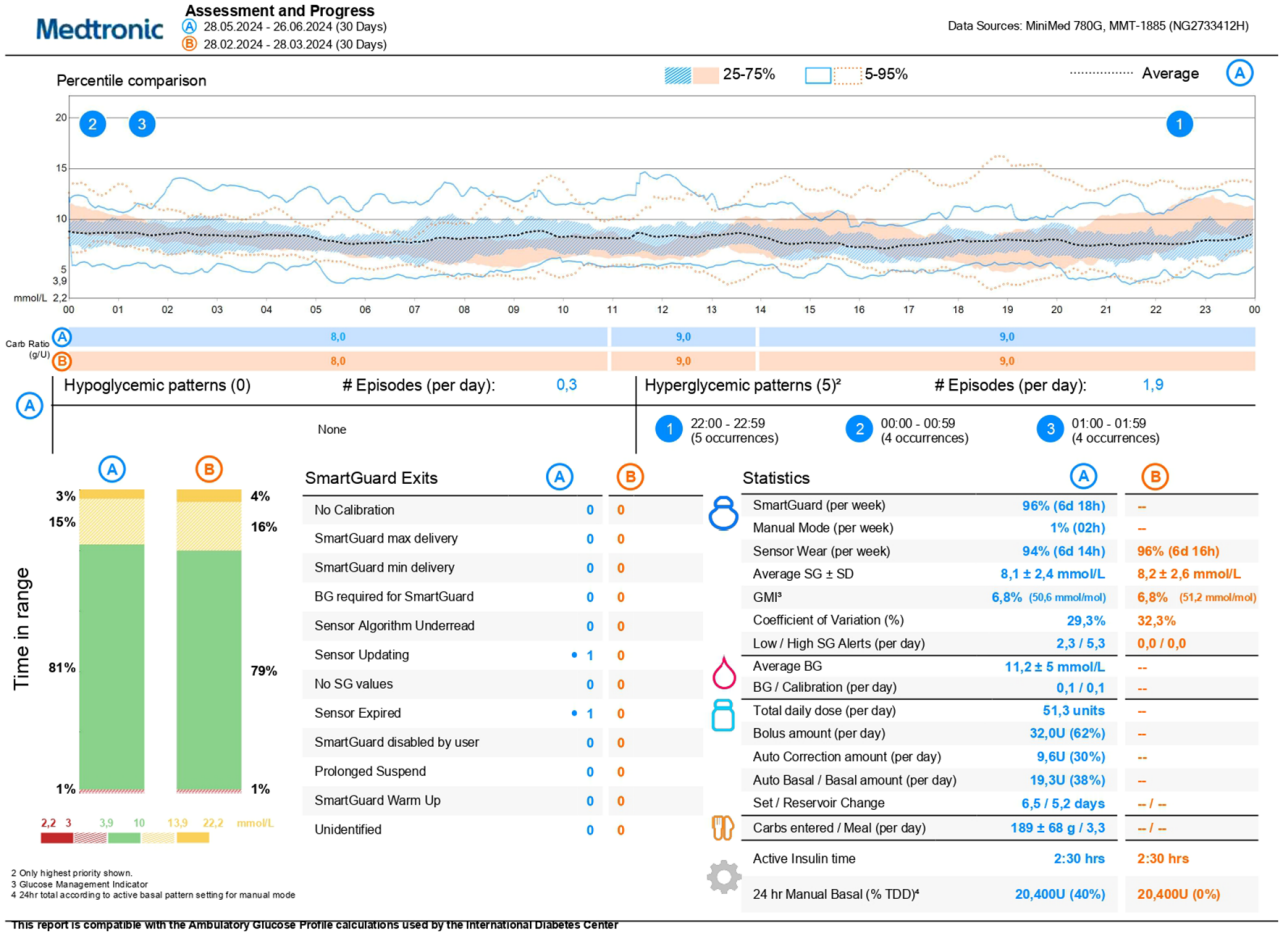
Current data from MiniMed 780 G insulin pump in June 2024, indicative of good glucose control and low time spent in hypoglycemia. HbA1c is 6.7%, time in range 81%, time below range 1%.

Shortly after the initiation of AID system, there was a significant increase in the time spent in target range (TIR) and a decrease in TBR, while maintaining the target HbA1c value (**Figure 2**). At the beginning of 2022, six months after starting the AID, the patient was retested for the hypoglycemia awareness with the results of Clarke score 2 and Gold score 2. Based on these results, his driving licence was reinstated. The patient has been treated with the MiniMed 780 G with satisfactory results to date (**Figure 3**), the restored hypoglycamia awareness persists, and since the initiation of the AID, there has been no episode of severe hypoglycemia.

The patient is currently satisfied with AID therapy and wishes to continue with it despite occasional technical complications. Considering the patient’s preference, we have maintained the SmartGuard target glucose at 6.7 mmol/L (120 mg/dL) and the active insulin time at 2:30. The improved awareness of hypoglycemia has brought significant psychological relief to the patient and his family, enabling a return to an active lifestyle, including physical activity and traveling.

## Discussion

There is a new possibility to improve impaired awareness of hypoglycemia using AID systems, even when all other recommended steps have failed. The improvement can be so significant and measurable that it may enable to restore a driving licence. This finding is consistent with a study conducted by Burckhardt et al. (3), where the use of AID led to a reduction in TBR and a significant improvement in hypoglycemia awareness in T1DM patients, as indicated by a decrease in the Gold score. The use of AID not only improves hypoglycemia awareness but also enhances sleep quality (6), glucose counterregulation, and autonomic symptoms (4). However, a study by Sherr et al. (7) found that even though the majority of participants with T1DM used CGM and half used AID, severe hypoglycemic events and impaired awareness of hypoglycemia still occurred. Therefore, the effect of advanced technologies is rather individual and there is an ongoing need for improved treatments strategies.

In addition to thorough education, modern insulin analogs, and higher target glucose levels, the use of CGM is crucial for managing impaired awareness of hypoglycemia (5, 8). In the case of our patient, we have done everything mentioned above. However, despite the patient’s compliance, prolonged hypoglycemia has not been eliminated. We acknowledge that initiating treatment with AID may not have been the only factor contributing to improved hypoglycemia awareness in our patient. The suspension of the patient’s driving license acted as a significant motivator for treatment adherence. Furthermore, the new technology may have enhanced motivation and facilitated better utilization of nutritional education and CGM-related training, which the patient underwent throughout the entire period.

Before the era of AID, in case of hypoglycemia unawareness, the only way to restore this complication was pancreas islet transplantation with consequent immunosuppression (9). Our patient met the criteria for the transplantation and was indicated for it, because the AID systems were just short time in use and there were no data for impaired awareness of hypoglycemia improvement. Fortunately, he decided not to undergo the transplantation and after initial reluctance he accepted the AID treatment.

The revocation of a driving licence is a very sensitive matter, as it significantly impacts the patient’s socioeconomic situation. This is also the reason why physicians often do not discuss this matter with the patient and do not investigate hypoglycemia unawareness. In our case, the revocation was taken based on a thorough examination of the patient’s individual case by a council of diabetologists. The exact criteria depend on local laws, but proven hypoglycemia unawareness is one of the conditions which limit the possibility of holding a driving licence (10). However, this step impacted our patient’s personal and professional life so significantly that he agreed to AID therapy, even though he initially did not want it. Data and evidence on how often the drivers licence in cases of improved hypoglycemia awareness is restored are still lacking.

## Data Availability

The original contributions presented in the study are included in the article/supplementary material. Further inquiries can be directed to the corresponding author.
